# Early administration of shenfu injection for the incidence of sepsis-induced cardiomyopathy in septic patients: a randomized controlled trial

**DOI:** 10.3389/fphar.2026.1682246

**Published:** 2026-02-27

**Authors:** Yaohui Liu, Yujie Fang, Shuliang Zhou, Yao Zhu, Jing Wang, Bo Hu

**Affiliations:** 1 Department of Critical Care Medicine, Zhongnan Hospital of Wuhan University, Wuhan, Hubei, China; 2 Clinical Research Center of Hubei Critical Care Medicine, Wuhan, Hubei, China

**Keywords:** myocardial injury biomarker, sepsis, sepsis-induced cardiomyopathy, septic shock, shenfu injection

## Abstract

**Background:**

Sepsis-induced cardiomyopathy (SIC) is a severe complication of sepsis that markedly increases mortality. Owing to its complex pathogenesis, no targeted drugs for SIC is currently available, highlighting the need for preventive interventions. This study aimed to evaluate whether early administration of Shenfu Injection (SFI) could prevent SIC.

**Methods:**

Patients diagnosed with sepsis 3.0 upon intensive care unit (ICU) admission but without SIC were randomly assigned to either the SFI group or control group via envelope randomization. The SFI group received intravenous SFI (200 mL/day) in addition to standard sepsis or septic shock management for 7 days (minimum 72 h if discontinued due to ICU transfer or death). The control group received an equivalent volume of saline alongside standard care. The primary outcome was the incidence of SIC within 7 days.

**Results:**

A total of 152 patients (76 per group) were analyzed. The incidence of SIC within 7 days was 9.2% in both groups. In the generalized linear mixed model (GLMM) adjusted for gender, age, septic shock and the baseline value of N-terminal pro-B-type natriuretic peptide (NT-proBNP), the interaction between time and group had a significant effect on NT-proBNP levels (P = 0.004). No significant differences were observed between groups in hemodynamic parameters, immune inflammatory indicators, organ function, vasoactive drug use, 7-day fluid balance, 28-day mortality, duration of mechanical ventilation, continuous renal replacement therapy duration, ICU stay, or total hospital stay.

**Conclusion:**

Early application of SFI did not significantly reduce the incidence of SIC in ICU patients with sepsis.

**Clinical Trial Registration:**

[https://www.chictr.org.cn/], identifier [ChiCTR2400088766].

## 1 Introduction

Sepsis is one of the most common critical illnesses encountered in the Intensive Care Unit (ICU), typically resulting from a dysregulated host response to infection and often leading to multiple organ dysfunction (American College of Chest Physicians, 1992; Levy et al., 2003; Rhodes et al., 2017). Sepsis-induced cardiomyopathy (SIC), first defined by Parker et al., in 1984, refers to a reversible myocardial depression syndrome caused by sepsis or septic shock (Parker et al., 1984). Epidemiological data suggest that approximately 10%–70% of patients with sepsis develop SIC (Beesley et al., 2018), and its occurrence is associated with a substantial increase in mortality, reaching as high as 70%–90% (Hasegawa et al., 2023). SIC is characterized by: (1) myocardial dysfunction induced by sepsis that is unrelated to pre-existing heart disease or myocardial ischemia; and (2) acute unilateral or bilateral myocardial systolic dysfunction, with or without diastolic dysfunction (Tsolaki et al., 2024; Zakynthinos et al., 2025). The diagnosis of SIC relies on a comprehensive assessment that includes echocardiography, myocardial injury markers, and hemodynamic parameters (Lukić et al., 2024). The most commonly employed echocardiographic parameter is left ventricular ejection fraction (LVEF) (Robotham et al., 1991; Beesley et al., 2018), while frequently used biomarkers of cardiac injury include high-sensitivity cardiac troponin I (hs-cTNI), creatine kinase-MB (CK-MB), and N-terminal pro-B-type natriuretic peptide (NT-proBNP) (Zakynthinos et al., 2025). In addition, superoxide dismutase (SOD) is often used to evaluate the degree of oxidative stress associated with sepsis-related myocardial injury (Xianchu et al., 2016). Other biomarkers such as heart-type fatty acid binding protein (h-FABP), human high mobility group protein B1 (HMGB1), and human histone H4 (H4) have also demonstrated diagnostic value in some studies (Oozawa et al., 2008; Zhang et al., 2012; Chen and Li, 2014; Kalbitz et al., 2015; Ye et al., 2018; Lu N. et al., 2019; Lu N.-F. et al., 2019; Jin et al., 2020; Weng et al., 2020; Ling et al., 2021; Wang et al., 2021; Wang L. et al., 2022).

The interplay between the cardiovascular, immune, autonomic nervous, metabolic, and microcirculation systems contributes to the complex pathogenesis and therapeutic challenges of SIC. The exact mechanisms underlying SIC remain incompletely understood, but known contributing factors include inflammatory response (Nabzdyk et al., 2019), oxidative stress (Ji et al., 2022), calcium balance disorder (Zhang and Liu, 2022), neuroendocrine disorder (Chen et al., 2022), immune imbalance (Fan et al., 2023), and microcirculation disorder (De Backer et al., 2014). Currently, no targeted drugs for SIC is currently available (Coopersmith, 2025). Management primarily focuses on controlling the underlying infection and providing organ support, including: (1) early source control with effective antimicrobial therapy; (2) fluid resuscitation and vasoactive agents (including cardiotonic drugs) to optimize hemodynamics; and (3) organ support such as mechanical ventilation (MV), continuous renal replacement therapy (CRRT), and extracorporeal membrane oxygenation (Padte et al., 2024). Therefore, developing pharmacological strategies to prevent SIC is of critical importance.

A large number of studies have explored the treatment of SIC with traditional Chinese medicine injections. The most common ones are Shenfu Injection (SFI) and Xuebijing Injection (According to the expert consensus on the integrated traditional Chinese and Western medicine treatment of SIC (2022)). Shenfu Injection (SFI) is derived from the botanical drugs hongshen and fuzi, with ginsenosides and aconitine as its principal active metabolites (Xu et al., 2024). SFI exerts multiple pharmacological effects, including enhancing myocardial energy metabolism (Zhao et al., 2025), reducing myocardial cell apoptosis (Yan et al., 2018; Chen et al., 2025), improving microcirculation and endothelial function (Wang S. et al., 2022; Tian et al., 2024), anti-inflammation activity (Huang et al., 2024), immune regulation (Zhang et al., 2017), alleviating mitochondrial damage (Xu et al., 2020), and improving hemodynamics (Li et al., 2022). SFI has been applied in the treatment of sepsis and cardiovascular diseases (Luo et al., 2021; Xu et al., 2024), it holds potential benefit for preventing SIC. There have been numerous literature reports on SIC treatment-related preclinical models and patient studies (Li et al., 2015; Wang et al., 2017; Huang et al., 2024; Yuan et al., 2025), with more clear efficacy data, deeper mechanisms, reliable safety. However, evidence regarding its preventive effect on SIC remains lacking. To address this gap, our research team conducted a prospective randomized controlled trial to investigate whether early administration of SFI could prevent SIC in adult ICU patients.

## 2 Materials and methods

### 2.1 Shenfu injection

We used the SFI (Approval number: Z51020664, Batch number: 230604AK01) manufactured by China Resources Sanjiu Pharmaceutical Co., Ltd., which is a metabolite of Panax ginseng C.A.Mey [Araliaceae; Ginseng radix et rhizoma rubra] and Aconitum carmichaelii Debx [Ranunculaceae; Aconiti lateralis radix praeparata] (Xu et al., 2024). Panax ginseng is sourced from Jilin Province, while Aconitum carmichaelii is from Sichuan Province, China, with the extraction ratio 2:1. The quality meets the standards (WS3-B-3427-98-2013) set by China Food and Drug Administration and it is achieved through fingerprinting and quantitative determination of characteristic component contents during producing. Voucher specimens were stored in the Plant Specimen Room of China Resources Sanjiu Pharmaceutical Co., Ltd. (File S1).

### 2.2 Research design

This was a prospective, randomized, placebo-controlled, single-center clinical trial. The study protocol was approved by the Ethics Committee of Zhongnan Hospital of Wuhan University (approval number: 2022096), in accordance with the Declaration of Helsinki and medical ethics standards. The trial was registered with the Chinese Clinical Trial Registry (Registration number: ChiCTR2400088766). All participants or their authorized representatives provided written informed consent. Our randomized controlled trail followed CONSORT 2010 guidelines to ensure rigor of the study (File S2).

### 2.3 Participants

This study was conducted in the Department of Critical Care Medicine at Zhongnan Hospital of Wuhan University, a top 40 tertiary grade-A hospital in China. The Inclusion, exclusion, and withdrawal criteria were as follows:

Inclusion criteria: Age ≥ 18 years old; Diagnosis of sepsis per Sepsis 3.0 criteria upon ICU admission.

Exclusion criteria: (1) Present with SIC upon admission to the ICU (referring to previous studies, SIC is defined as sepsis patients excluding acute coronary syndrome and stress cardiomyopathy, with left ventricular dysfunction (LVEF < 50%) and hs-cTNI higher than twice the upper limit of the normal value); (2) Patients with a history of allergy to SFI (including any metabolites of the botanical ingredients or excipients in the formulation), or patients with contraindications to SFI (with a history of severe adverse reactions such as anaphylactic shock or severe dyspnea); (3) Pregnant women; (4) Patients who are unable to complete bedside cardiac ultrasound examinations for various reasons; (5) Patients after cardiac surgery; (6) The patient participated in another clinical study simultaneously; (7) The patient has severe underlying heart diseases such as dilated heart disease, valvular heart disease, ventricular septal defect, congenital heart disease, etc. (8) Patients who are expected to die within 48 h; (9) Patients who refuse to participate.

Withdrawal criteria: (1) Those who withdraw their informed consent; (2) Those whose ICU stay is less than 72 h; (3) Those whose treatment plans need to be changed due to the aggravation of their conditions.

### 2.4 Randomization

The Clinical Research Department of Zhongnan Hospital implemented block randomization. Statisticians predefined the seed number in the statistical software to generate random sequences, employing an envelope method for allocation concealment. Each patient’s group assignment was sealed in an opaque envelope, inaccessible from the outside. Designated personnel maintained the randomization list and envelopes. After eligibility confirmation, researchers opened the envelopes in order of patient enrollment to reveal and record group assignment.

### 2.5 Sample size calculation

Previous studies have shown that the incidence of septic cardiomyopathy is approximately 60% (Landesberg et al., 2012). Due to the lack of clinical research on the prevention of septic cardiomyopathy by Shenfu Injection, based on the previous research results of Shenfu Injection in the treatment of septic cardiomyopathy, it is estimated that Shenfu Injection can reduce the incidence of septic cardiomyopathy by 30%. Moreover, the incidence of septic cardiomyopathy in the Shenfu group was 5% lower than that in the control group, which can be regarded as having clinical significance. A type of error α was set at 0.05 (two-sided), and the certainty (1-β) was set at 90%. Using the hypothesis test calculation formula for two-sample rate comparison, it was determined that the sample size of each group was 76 cases. Calculated at a dropout rate of 10%, the total sample size of this study was finally 168 cases.

### 2.6 Interventions

All patients received standard treatment per the 2021 International Guidelines for the Management of Sepsis and Septic Shock (Evans et al., 2021), including antimicrobial therapy, fluid resuscitation, vasoactive drugs, MV, sedation, analgesia, and CRRT as needed. In the SFI group, patients received continuous intravenous infusion of SFI (100 mL twice daily, 20 mL/h) for 7 consecutive days. If discontinued early due to ICU transfer or death, a minimum treatment duration of 72 h was required. The control group received an equivalent volume and rate of normal saline under the same treatment protocol.

### 2.7 Measurements

Based on the different potential onset time points of SIC, our research team defined the first 72 h after admission to the ICU as the early onset stage (T0), days 4–5 as the middle onset stage (T1), and days 6–7 as the late onset stage (T2).

Baseline data: On the day of enrollment, the patient’s age, gender, body mass index (BMI), source of infection, underlying diseases, acute physiology and chronic health evaluation II (APACHE II) score, sequential organ failure assessment (SOFA) score, organ function involvement, proportion of MV and CRRT, and the use of vasoactive drugs were recorded. (Including the proportion of norepinephrine (NE) usage, the proportion of vasoactive drug usage, the 2021 version of the vasoactive-inotropic score (VIS score), and noradrenaline equivalent (NEE) (for the calculation method, please refer to Supplementary File S1). (2) Ultrasound indicators: Bedside cardiac ultrasound screening for LVEF. (3) Myocardial injury markers: NT-proBNP, CK-MB, hs-cTNI, SOD, h-FABP, HMGB1, H4. (4) Hemodynamic parameters: heart rate (HR), mean arterial pressure (MAP), central venous pressure (CVP), pulse pressure (PP), mean perfusion pressure (MPP), coronary perfusion pressure (CPP), serum lactate concentration (lac), lactate clearance rate (LCR), central venous oxygen saturation (ScvO2), central venous-to-arterial carbon dioxide difference (GAP). (5) Vasoactive drug usage: Record the usage of all vasoactive drugs and calculate the VIS score 2021 version and NEE. (6) Total fluid balance volume. (7) Immune inflammatory indicators: Procalcitonin (PCT), neutrophil (NEUT), lymphocyte (LYM), ; procalcitonin clearance (PCTc), ; neutrophil-to- lymphocyte ratio (NLR), platelet-to- lymphocyte rate (PLR), neutrophil/(leukocyte minus lymphocyte) (dNLR), systemic immune-inflammation (platelet* neutrophil/lymphocyte) (SII), (8) Organ functions: platelet (PLT), (B) total bilirubin (TB), aspartic transaminase (AST), creatinine(CREA), urea nitrogen(BUN). (9) Absolute counts of lymphocyte subsets (T lymphocytes, helper/inducer T lymphocytes, suppressor/cytotoxic T lymphocytes, B lymphocytes, NK cells) on the 3rd to 4th day of enrollment. (10) Safety indicators: Statistically analyze the types and incidence rates of common adverse reactions (arrhythmia, allergic reactions, Shenfu-related neurological abnormalities, and Shenfu-related liver injury) of SFI within 7 days.

The primary outcome of the study was the incidence of SIC within 7 days after enrollment. The diagnostic criteria for SIC in this study were: Patients with sepsis demonstrated left ventricular systolic dysfunction (LVEF < 50%) on echocardiography and elevated hs-cTnI was twice the upper limit of the normal value (the upper limit of the normal value of the reference range of the hs-cTNI kit used in this study was 26.2 pg/mL), after ruling out acute coronary syndrome and stress-induced cardiomyopathy.

Secondary outcome include: LVEF, myocardial injury markers (hs-cTNI, NT-proBNP, CK MB, SOD, HMGB 1. H4, h-FABP), hemodynamic parameters (HR, MAP, PP, CVP, MPP, CPP, GAP, SCVO2, Lac, LCR), use of vasoactive drugs (VIS score 2021 edition, NEE), fluid balance volume of the previous week, immune inflammatory indicators (PCT, PCTc, N, L, NLR, PLR, dNLR, SII), organ function indicators (PLT, AST, TB, Cr, BUN), safety indicators (types and incidence of common adverse reactions of SFI within 7 days (including arrhythmia, allergic reaction, Shenfu related neurological abnormality, Shenfu related liver injury)), prognosis indicators (28-day mortality, ICU length of stay (ICU-LOS), hospital length of stay(H-LOS), MV time, CRRT time).

### 2.8 Statistical analysis

Measurement data were tested for normality using the Shapiro-Wilk test or Kolmogorov-Smirnov test. Data that conformed to the normal distribution or approximately conformed to the normal distribution were expressed as mean ± standard deviation. Independent sample t-test was used for inter-group analysis. Data with skewed distribution were expressed as median (interquartile ranges). The Mann-Whitney U test was used for comparison between groups. Count data were expressed as percentages, and the analysis between groups was performed using the χ2 test or Fisher’s exact probability method. Based on previous studies and the Directed Acyclic Graph (File S3 Supplementary Figure S1), we identified the minimum covariates as gender, age, and septic shock. For the repeated-measurements of myocardial injury biomarkers and hemodynamic parameters, we used the generalized linear mixed-effects model or the mixed-effects model to correct for the minimum covariates and baseline values, and then compared the differences between the groups. Survival rates were studied using the Kaplan-Meier method and compared using the log-rank test. Binary logistic regression analysis was conducted to perform subgroup analysis on the 28-day mortality rate, and a forest plot was generated to visually display the estimated effect values for each subgroup. Interaction terms between the exposure variables and group variable were included in the logistic regression model to assess potential effect modification. Based on the previous research results, *post hoc* subgroup analysis was conducted with VIS score of 15 points, NEE of 0.1mcg/kg/min, and lactic acid concentration of 2.0 mmol/L as the cut-off values to avoid the influence of individual differences on the analysis results,. When analyzing the primary outcome, it was conducted separately based on whether the patients had septic shock at the time of enrollment. When analyzing the length of stay in the ICU and the total length of stay, the patients were divided into the survival group and the death group for separate analysis. Date analysis was performed using SPSS 29.0.1.0 and R software (version 4.2.2), along with the use of MSTATA software (https://www.mstata.com/). All P values were bilateral, and a P value <0.05 was considered statistically significant.

## 3 Results

### 3.1 Participant recruitment

**FIGURE 1:**
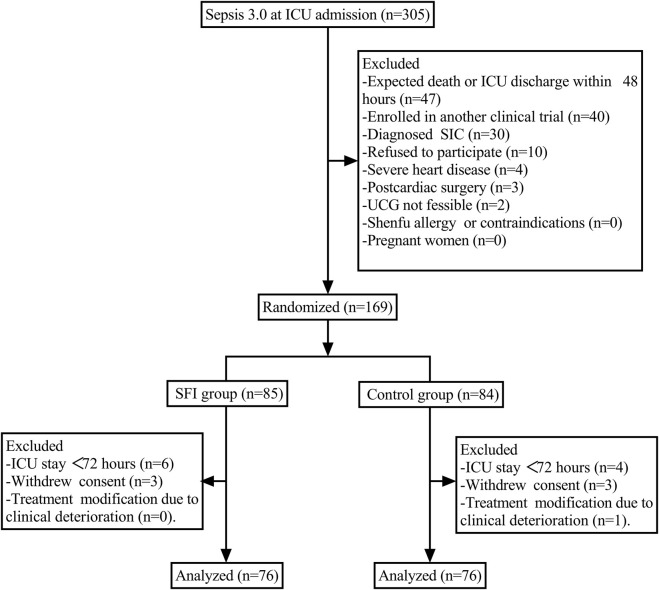
Flow chart of the study. Notes: Abbreviations: ICU, Intensive Care Unit; UCG, Ultrasound Cardiogram; SFI, Shenfu Injection.

From August 2023 to January 2025, a total of 305 patients admitted to the ICU who met the Sepsis 3.0 diagnostic criteria were initially screened for eligibility. Based on the exclusion criteria, 136 patients were excluded, leaving 169 patients who underwent randomization. Subsequently, 17 patients were excluded according to the predefined withdrawal criteria. Ultimately, 76 patients in the Shenfu group and 76 patients in the control group were included in the final statistical analysis (Figure 1).

### 3.2 Baseline characteristics

The average age of patients in the SFI group was 63.34 years, with 57.9% being male, and a mean BMI of 22.38. In the control group, the average age was 62.62 years, 68.4% were male, and the mean BMI was 23.22. The baseline characteristics between the two groups were generally comparable. The most common comorbidities in both groups were hypertension, diabetes, and malignant tumors. The majority of infections were community-acquired, with the lungs being the most frequently involved site. Gram-negative bacilli were the predominant pathogens identified in both groups. There were no significant differences between groups in disease severity, as reflected by APACHE II and SOFA scores, or in the proportion of patients with septic shock. The primary organs affected included the respiratory, nervous, and circulatory systems. Additionally, no significant differences were observed between the two groups in baseline hemodynamic parameters, immune-inflammatory markers, or organ function indicators (Table 1).

**TABLE 1:** Baseline characteristics of patients from the SFI group and control group.

Variable	Total (n = 152)	SFI group (n = 76)	Control group (n = 76)	*SMD*	*95% CI*
Demographical					
Age (years)	62.98 ± 14.67	63.34 ± 14.18	62.62 ± 15.23	0.05	−0.27–0.37
Male	96(63.2)	44(57.9)	52(68.4)	−0.22	−0.54–0.10
BMI (kg/m^2)	22.80 ± 3.58	22.38 ± 3.58	23.22 ± 3.55	−0.24	−0.56–0.08
Medical history					
Hypertension	68(44.7)	30(39.5)	38(50.0)	−0.21	−0.53–0.11
Diabetes	42(27.6)	20(26.3)	22(28.9)	−0.06	−0.38–0.26
CHD	24(15.8)	10(13.2)	14(18.4)	−0.14	−0.46–0.17
Malignant tumor	41(27.0)	20(26.3)	21(27.6)	−0.03	−0.35–0.29
Stroke	26(17.1)	12(15.8)	14(18.4)	−0.07	−0.39–0.25
CKD	17(11.2)	7(9.2)	10(13.2)	−0.13	−0.44–0.19
COPD	9(5.9)	6(7.9)	3(3.9)	0.17	−0.15–0.49
Community-acquired	87(57.2)	45(59.2)	42(55.3)	0.08	−0.24–0.40
Site of infection					
Lung	95(62.5)	44(57.9)	51(67.1)	−0.19	−0.51–0.13
Urinary tract	28(18.4)	15(19.7)	13(17.1)	0.07	−0.25–0.39
Abdominal cavity	38(25.0)	21(27.6)	17(22.4)	0.12	−0.20–0.44
Hepatobiliary system	22(14.5)	11(14.5)	11(14.5)	0.00	−0.32–0.32
Bloodstream	27(17.8)	14(18.4)	13(17.1)	0.03	−0.28–0.35
Pathogen					
GNB	56(36.8)	29(38.2)	27(35.5)	0.05	−0.26–0.37
GPB	15(9.9)	5(6.6)	10(13.2)	−0.22	−0.54–0.10
GPB/GNB	22(14.5)	11(14.5)	11(14.5)	0.00	−0.32–0.32
Fungi	24(15.8)	10(13.2)	14(18.4)	−0.14	−0.46–0.17
Virus	24(15.8)	8(10.5)	16(21.1)	−0.29	−0.61–0.03
Scores					
APACHE II	22.93 ± 6.07	23.21 ± 6.48	22.66 ± 5.67	0.09	−0.23–0.41
SOFA	9.89 ± 3.57	9.74 ± 3.53	10.05 ± 3.62	−0.09	−0.41–0.23
Organ dysfunction					
Neurological	110(72.4)	53(69.7)	57(75.0)	−0.12	−0.44–0.20
Respiratory	143(94.1)	72(94.7)	71(93.4)	0.06	−0.26–0.37
Circulatory	125(82.2)	66(86.8)	59(77.6)	0.24	−0.08–0.56
Hepatic	77(50.7)	40(52.6)	37(48.7)	0.08	−0.24–0.40
Renal	75(49.3)	37(48.7)	38(50.0)	−0.03	−0.34–0.29
Coagulation	95(62.5)	46(60.5)	49(64.5)	−0.08	−0.40–0.24
Septic shock	75(49.3)	43(56.6)	32(42.1)	0.29	−0.03–0.61
Hemodynamic parameters					
LVEF (%)	60.19 ± 9.58	59.84 ± 9.36	60.54 ± 9.84	−0.07	−0.39–0.24
HR (bpm)	89.51 ± 17.87	88.53 ± 18.24	90.50 ± 17.55	−0.11	−0.43–0.21
SBP (mmHg)	127.43 ± 21.44	127.86 ± 19.45	127.00 ± 23.37	0.04	−0.28–0.36
DBP (mmHg)	62.93 ± 10.68	62.21 ± 9.98	63.66 ± 11.35	−0.14	−0.45–0.18
CVP (mmHg)	9.31 ± 3.39	9.52 ± 3.58	9.09 ± 3.21	0.14	−0.27–0.55
Lac (mmol/L)	1.85(1.10,3.28)	2.15(1.23,3.70)	1.65(1.10,2.98)	0.32	0.00–0.64
Laboratory test indicators					
PCT (ng/mL)	11.80(2.40,59.70)	14.16(3.13,63.32)	10.34(1.44,46.89)	0.10	−0.21–0.42
NEUT (10^9/L)	10.45(5.94,16.61)	10.61(6.08,18.45)	10.45(5.44,16.34)	0.07	−0.25–0.39
LYM (10^9/L)	0.58(0.26,0.88)	0.51(0.23,0.85)	0.63(0.30,0.89)	−0.03	−0.35–0.28
TB (μmol/L)	20.90(11.30,38.40)	22.50(11.30,39.40)	19.40(11.15,36.53)	0.11	−0.21–0.43
AST (U/L)	55.00(29.00,127.25)	55.00(31.50,137.00)	55.00(28.00,126.00)	0.15	−0.17–0.46
CREA (μmol/L)	113.25(72.28,272.38)	112.75(73.20,227.63)	113.45(67.25,293.13)	−0.09	−0.41–0.23
BUN (mmol/L)	12.53(7.90,22.10)	12.63.(8.19,22.07)	12.00(7.20,22.98)	−0.11	−0.42–0.21
PLT (10^9/L)	129.00(79.00,197.00)	144.50(76.00,215.75)	123.00(73.25,190.75)	0.15	−0.17–0.47
T cells (10^6/L)	307.00(182.00,643.00)	341.00(196.00,649.00)	290.50(175.50,642.75)	0.14	−0.20–0.48
Th (10^6/L)	206.00(115.00,349.00)	238.00(116.00,363.00)	202.50(107.75,345.50)	0.13	−0.20–0.47
Tc (10^6/L)	84.00(45.00,180.00)	78.00(47.00,189.00)	86.00(43.75,160.75)	0.11	−0.23–0.44
B cells (10^6/L)	78.00(37.00,156.00)	81.00(50.00,157.00)	70.50(26.25,157.75)	0.24	−0.09–0.58
NK (10^6/L)	50.00(28.00,105.50)	43.00(24.00,107.00)	53.00(35.00,105.25)	0.04	−0.29–0.38
Others					
Vasoactive agents	95(62.5)	51(67.1)	44(57.9)	0.19	−0.13–0.51
NE	67(44.1)	38(50.0)	29(38.2)	0.24	−0.08–0.56
MV	105(69.1)	52(68.4)	53(69.7)	−0.03	−0.35–0.29
CRRT	23(15.1)	11(14.5)	12(15.8)	−0.04	−0.35–0.28

Continuous data were presented as mean (SD) or median (IQR). Categorical data are presented as counts (%).

Abbreviations: BMI, body mass index; CHD, coronary heart disease; CKD, chronic kidney diseases; COPD, chronic obstructive pulmonary disease; GNB, Gram-negative bacterium; GPB, Gram positive bacteria; GPB/GNB, Gram positive bacteria and Gram-negative bacterium; APACHE II, acute physiology and chronic health evaluation II; SOFA, sequential organ failure assessment; LVEF, left ventricular ejection fraction; HR, heart rate; SBP, systolic blood pressure; DBP, diastolic blood pressure; CVP, central venous pressure; lac, lactic acid; NE, norepinephrine; MV, mechanical ventilation; CRRT, continuous renal replacement therapy; PCT, procalcitonin; NEUT, neutrophil; LYM, lymphocyte; TB, total bilirubin; AST, aspartic transaminase; CREA, creatinine; BUN, urea nitrogen; PLT, platelet; T cells, T lymphocytes; Th, helper T lymphocytes; Tc, cytotoxic T lymphocytes; B cells, B lymphocytes; NK, natural killer cell.

### 3.3 Primary outcome

In this study, SIC was diagnosed based on a combination of LVEF and hs-cTNI assessments. The overall incidence of SIC within 7 days after enrollment was 9.2% in both the SFI group and the control group, with no significant difference in the incidence between the groups at any time point or the recovery rate of SIC within 7 days. The onset of SIC predominantly occurred within the first 3 days following enrollment. The same conclusion was also drawn in the subgroup analysis of patients with septic shock (Table 2).

**TABLE 2:** The incidence of SIC in both groups of patients at different time periods.

Variable	All patients	SFI group	Control group	*P value*	SMD	*95% CI*
The incidence of SIC in all patients						
Incidence (total)	9.2%	9.2%	9.2%	1.000	0.00	−0.32–0.32
Incidence (T0)	7.2%	5.3%	9.2%	0.348	−0.15	−0.47–0.17
Incidence (T1)	2.0%	3.9%	0	0.244	0.29	−0.03–0.61
Incidence (T2)	0	0	0	​	​	​
Recovered within 7 days	64.3%	71.4%	57.1%	1.000	0.30	−0.75–1.36
The incidence of SIC in the septic shock subgroup						
Incidence (total)	16.0%	14.0%	18.8%	0.575	−0.13	−0.59–0.33
Incidence (T0)	12.0%	7.0%	18.8%	0.121	−0.36	−0.82–0.10
Incidence (T1)	4.0%	7.0%	0	0.353	0.39	−0.07–0.85
Incidence (T2)	0	0	0	​	​	​
Recovered within 7 days	58.3%	66.7%	50.0%	1.000	0.34	−0.80–1.48

Abbreviations: SFI, shenfu injection; SIC, sepsis-induced cardiomyopathy.

### 3.4 Secondary outcome

#### 3.4.1 LVEF and biomarkers of myocardial injury

**FIGURE 2:**
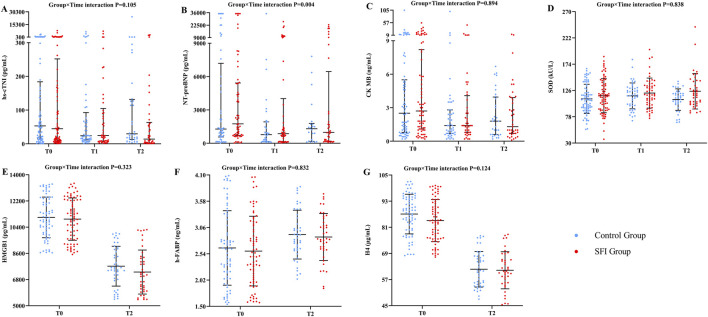
The differences in biomarkers of myocardial injury at different times between two groups. Variable were presented as mean (SD) or median (IQR). **(A)** hs-cTNI, high-sensitivity cardiac troponin I; **(B)** NT-proBNP, N-terminal pro-B-type natriuretic peptide; **(C)** CKMB, creatine kinase-MB; **(D)** SOD, superoxide dismutase; **(E)** HMGB 1, human high mobility group protein B1; **(F)** h-FABP, heart-type fatty acid binding protein; **(G)** Human H4, human histone H4. *indicates significant differences (P < 0.05).

There was no significant difference in LVEF between the SFI group and the control group at any of the assessed time points (Table 3). In the GLMM adjusted for gender, age, septic shock, and the baseline value of N-terminal pro-brain natriuretic peptide (NT-proBNP), the interaction between time and group had a significant effect on NT-proBNP levels (P = 0.004). No significant differences were observed in other myocardial injury biomarkers (Figure 2; Supplementary Tables S2-S9 in File S3).

**TABLE 3:** Comparison of LVEF values at different time periods between two groups of patients.

Time	SFI group	Control group	*SMD*	*95% CI*
T0	59.84 ± 9.36	60.54 ± 9.84	−0.07	−0.39–0.24
T1	63.18 ± 8.84	64.07 ± 8.02	−0.11	−0.42–0.21
T2	64.36 ± 9.07	66.14 ± 8.97	−0.20	−0.58–0.19

Continuous data were presented as mean (SD).

#### 3.4.2 Hemodynamic parameters and liquid equilibrium volume

**FIGURE 3:**
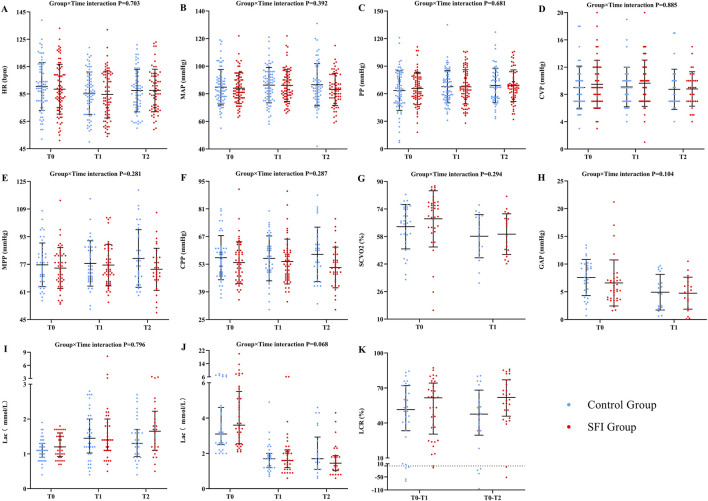
The differences in hemodynamic parameters at different times between two groups. Variable were presented as mean (SD) or median (IQR). **(A)** HR, heart rate; **(B)** MAP, mean arterial pressure; **(C)** PP, pulse pressure; **(D)** CVP, central venous pressure; **(E)** MPP; mean perfusion pressure; **(F)** CPP, coronary perfusion pressure; **(G)** SCVO2, central venous oxygen saturation; **(H)** GAP, central venous-to-arterial carbon dioxide difference; **(I)** lac(lactic acid)<2 mmol/L; **(J)** lac≥2 mmol/L; **(K)** LCR, lactate clearance. *indicates significant differences (P < 0.05).

At T0, there were no significant differences in hemodynamic parameters between the two groups. After adjusting for gender, age, septic shock and baseline hemodynamic parameters, the LMM or GLMM was conducted to analyze the interaction effect of time and group on each hemodynamic parameter. The results showed no significant differences. Although there was a statistically significant difference in LCR between the two groups, the 95% CI of the effect size estimate crossed the invalid value of 0. The effect size estimate was uncertain and no significant difference between the groups could be determined (P = 0.028; SMD = 0.56, 95% CI: 0.01–1.13) (Figure 3; Supplementary Tables S10-S20 in File S3). The cumulative fluid balance over 7 days did not differ significantly between the SFI group (1804.98 ± 3124.74 mL) and the control group (1527.31 ± 3928.21 mL) (P = 0.697).

#### 3.4.3 Vasoactive agent utilization

At T0, there were no significant differences in the use of vasoactive drugs across all subgroups. In the subgroup with VIS <15, the VIS score at T1 in the SFI group was significantly higher than that in the control group. However, the median absolute values of VIS and NEEwere both zero, and there was no significant differences in the magnitude of change from T0. Futhermore, in the subgroup with NEE <0.1 μg/kg/min, the effect size analysis of t1, SFI showed a moderate effect on NEE, but there was no significant difference. At T2, the decrease in NEE in the SFI group compared to T0 was statistically different from that in the control group. However, the effect size analysis was negative, and no significant difference between the groups could be determined (Table 4).

**TABLE 4:** Hierarchical analysis of the vasoactive agent utilization.

Variable	SFI group	Control group	*P value*	SMD	*95% CI*
VIS < 15 subgroup					
VIS (T0)^1^	0.00 (0.00,4.63)	0.00 (0.00,0.00)	0.266	0.26	−0.11–0.63
VIS (T1)^2^	0.00 (0.00,5.79)	0.00 (0.00,0.00)	0.003	0.55	0.16–0.93
VIS (T2)^3^	0.00 (0.00,4.63)	0.00 (0.00,0.00)	0.118	0.20	−0.23–0.64
VIS (T0-T1)^2^	0.00 (−2.31,0.00)	0.00 (0.00,0.00)	0.132	−0.45	−0.83–0.07
VIS (T0-T2)^3^	0.00 (0.00,1.16)	0.00 (0.00,0.00)	0.974	−0.10	−0.54–0.34
VIS > 15 subgroup					
VIS (T0)^4^	52.04 (27.78,80.38)	41.67 (27.78,62.50)	0.638	−0.12	−0.76–0.53
VIS (T1)^5^	0.00 (0.00,18.52)	0.00 (0.00,11.57)	0.897	0.23	−0.45–0.92
VIS (T2)^6^	0.00 (0.00,13.89)	0.00 (0.00,6.94)	0.933	0.54	−0.22–1.29
VIS (T1-T0)^5^	45.14 (18.52,69.44)	41.67 (20.83,53.24)	0.795	−0.05	−0.73–0.63
VIS (T2-T0)^6^	45.14 (18.52,69.44)	41.67 (22.08,53.01)	0.933	0.54	−0.21–1.30
NEE < 0.1 subgroup					
NEE (T0)^7^	0.00 (0.00,0.05)	0.00 (0.00,0.05)	0.527	0.15	−0.27–0.56
NEE (T1)^8^	0.00 (0.00,0.10)	0.00 (0.00,0.00)	0.113	0.53	0.10–0.95
NEE (T2)^9^	0.00 (0.00,0.09)	0.00 (0.00,0.00)	0.044	0.37	−0.12–0.85
NEE (T0-T1)^8^	0.00 (−0.10,0.01)	0.00 (0.00,0.01)	0.260	−0.43	−0.85–0.01
NEE (T0-T2)^9^	0.00 (−0.09,0.03)	0.00 (0.00,0.03)	0.272	−0.29	−0.77–0.20
NEE > 0.1 subgroup					
NEE (T0)^10^	0.36 (0.19,0.66)	0.24 (0.14,0.47)	0.055	0.12	−0.38–0.62
NEE (T1)^11^	0.00 (0.00,0.19)	0.00 (0.00,0.08)	0.174	0.39	−0.13–0.92
NEE (T2)^12^	0.00 (0.00,0.14)	0.00 (0.00,0.12)	0.422	0.25	−0.34–0.83
NEE (T0-T1)^11^	0.30 (0.14,0.52)	0.16 (0.14,0.44)	0.259	0.11	−0.41–0.64
NEE (T0-T2)^12^	0.30 (0.19,0.63)	0.15 (0.05,0.42)	0.100	0.22	−0.36–0.81

Continuous data were presented as median (IQR). 1:SFI, group n = 52; Control group n = 61; 2:SFI, group n = 50; Control group n = 59; 3:SFI, group n = 37; Control group n = 44; 4:SFI, group n = 24; Control group n = 15; 5:SFI, group n = 23; Control group n = 13; 6:SFI, group n = 19; Control group n = 11; 7:SFI, group n = :42; Control group n = 48; 8:SFI, group n = :42; Control group n = 47; 9:SFI, group n = 33; Control group n = 33; 10:SFI, group n = 34; Control group n = 28; 11:SFI, group n = 32; Control group n = 25; 12:SFI, group n = 23; Control group n = 22.

Abbreviations: VIS, version of the vasoactive-inotropic score; NEE, noradrenaline equivalent.

#### 3.4.4 Immune inflammatory indicators

**FIGURE 4:**
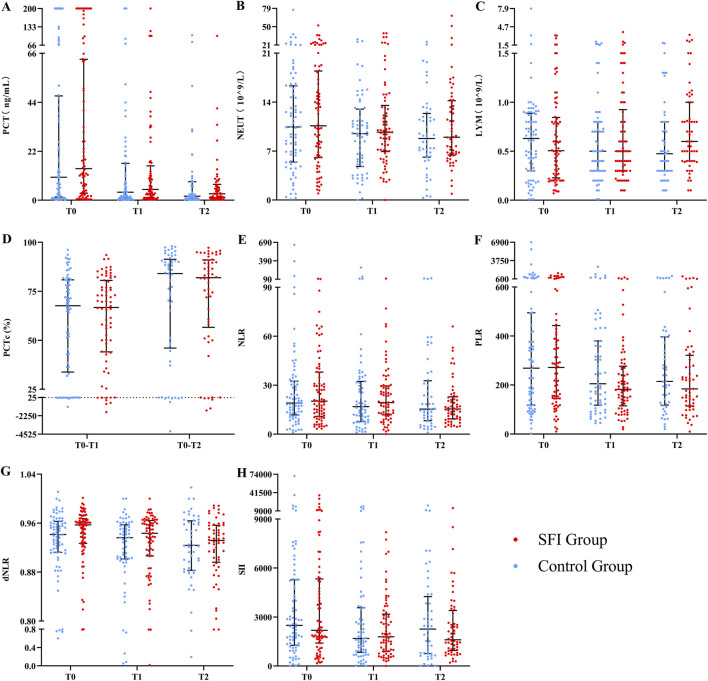
The differences in immune inflammatory indicators at different times between two groups. Variable were presented as mean (SD) or median (IQR). **(A)** PCT, procalcitonin; **(B)** NEUT, neutrophil; **(C)** LYM, lymphocyte; **(D)** PCTc, procalcitonin clearance; **(E)** NLR, neutrophil-to- lymphocyte ratio; **(F)** PLR, platelet-to- lymphocyte rate; **(G)** dNLR, neutrophil/(leukocyte minus lymphocyte); **(H)** SII systemic immune-inflammation (platelet* neutrophil/lymphocyte). *indicates significant differences (P < 0.05).

At T2, There were statistically significant differences in LYM among the groups, but the effect size estimate’s 95% CI crossed 0. As a result, the outcome was uncertain and could not reflect a significant difference between the groups (0 = 0.047, SMD = 0.36, 95% CI: 0.03–0.75). At all other time points, no significant differences were observed between the groups in PCT, PCTc, N, NLR, PLR, dNLR, or SII (Figure 4).

#### 3.4.5 Organ function

**FIGURE 5:**
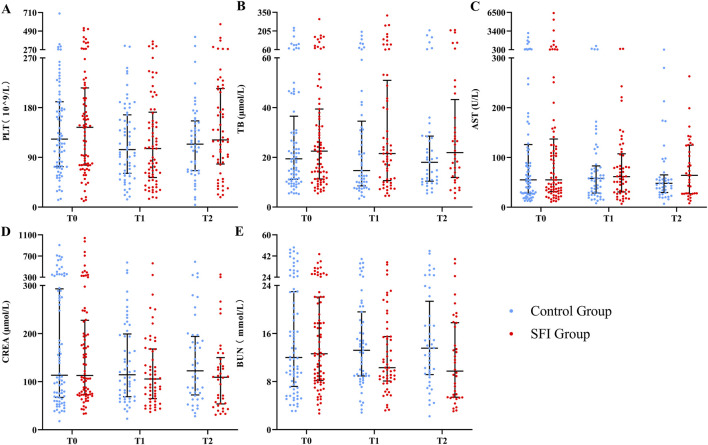
The differences in organ function at different times between two groups. Variable were presented as mean (SD) or median (IQR). **(A)** PLT, platelet; **(B)** TB, total bilirubin; **(C)** AST, aspartic transaminase; **(D)** CREA, creatinine; **(E)** BUN, urea nitrogen. *indicates significant differences (P < 0.05).

At T0, liver and kidney function indicators, as well as platelet counts, were comparable between the two groups. At T2, There were statistically significant differences in BUNamong the groups, but the effect size estimate’s 95% CI crossed 0. As a result, the outcome was uncertain and could not reflect a significant difference between the groups (0 = 0.047, SMD = −0.40, 95% CI: 0.84–0.04). No significant differences were observed between the groups in PLT, TB, AST, BUN or CREA at any other time point (Figure 5).

#### 3.4.6 Prognostic indicator

**FIGURE 6:**
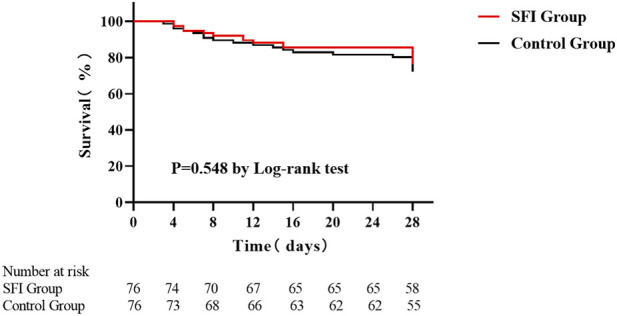
Kaplan-Meier survival curves.

During the 28-day follow-up period, the mortality rate was 23.7% in the SFI group and 27.6% in the control group. In total, 39 patients died across both groups, resulting in an overall mortality rate of 25.7%. There was no significant difference in 28-day mortality between the two groups. The Kaplan-Meier survival curves are presented in Figure 6. Subgroup analysis and interaction test for 28-day mortality also both showed negative results (Supplementary Figure S2 of Supplementary File S3). Additionally, no significant differences were observed between the groups in ICU-LOS, H-LOS, the proportion of patients requiring MV or CRRT, or in the duration of MV and CRRT (Table 5).

**TABLE 5:** Comparison of prognostic indicators between the two groups of patients.

Variable	SFI group	Control group	P value	SMD	95% CI
28-day mortality	18(23.7)	21(27.6)	0.577	−0.09	−0.41–0.23
Survival group ICU-LOS (d)	8.50(5.75,14.00)	9.00(5.00,14.00)	0.940	0.01	−0.36–0.38
Survival group H-LOS (d)	16.00(11.00,32.25)	17.00(11.00,24.00)	0.794	0.09	−0.28–0.46
Death group ICU-LOS (d)	7.50(4.75,11.25)	10.00(5.00,16.50)	0.410	−0.35	−0.98–0.28
Death group H-LOS (d)	11.50(7.00,17.00)	17.00(10.00,24.50)	0.053	−0.78	−1.44–0.13
MV (n, %)	68(89.5)	66(86.8)	0.616	0.08	−0.24–0.40
CRRT (n, %)	30(39.5)	30(39.5)	1.000	0.00	−0.32–0.32
MV (h)	154.50(93.50,237.25)	153.50(85.00,290.25)	0.972	0.12	−0.22–0.46
CRRT (h)	73.00(63.00,154.00)	74.00(52.75,150.00)	0.960	−0.12	−0.63–0.38

Continuous data were presented as median (IQR). Categorical data are presented as counts (%).

Abbreviations: SFI, shenfu injection, ICU-LOS ICU, length of stay, H-LOS, hospital length of stay; MV, mechanical ventilation; CRRT, continuous renal replacement therapy.

#### 3.4.7 Safety

The incidence and types of arrhythmia within 7 days were comparable between the SFI and control groups. The most frequently observed arrhythmias were sinus tachycardia and premature atrial contractions. The effect size analysis indicated that the use of Shenfu Injection had a small effect on the incidence of atrial tachycardia, but the difference was not statistically significant. No significant diferences between the groups for other types of arrhythmias. There was also no significant difference between the groups in the proportion of patients receiving antiarrhythmic medications (Table 6). No other adverse reactions were reported in either group, including allergic reactions, shenfu-related neurofunctional abnormalities, or shenfu-related liver injury.

**TABLE 6:** Comparison table of arrhythmia types and incidence between two groups.

Variable	SFI group (n = 76)	Control group (n = 76)	P value	SMD	95% CI
Arrhythmia	58(76.3)	59(77.6)	0.847	−0.03	−0.35–0.29
Antiarrhythmic drugs	38(50.0)	36(47.4)	0.746	0.05	−0.27–0.37
Nodal tachycardia	38(50.0)	44(57.9)	0.329	−0.16	−0.48–0.16
Atrial premature complex	14(18.4)	14(18.4)	1.000	0.00	−0.32–0.32
Atrial fibrillation	8(10.5)	8(10.5)	1.000	0.00	−0.32–0.32
Incomplete right bundle branch block	7(9.2)	6(7.9)	0.772	0.05	−0.27–0.37
Ventricular premature complex	6(7.9)	7(9.2)	0.772	−0.05	−0.37–0.27
Atrial flutter	4(5.3)	1(1.3)	0.363	0.22	−0.10–0.54
Atrial tachycardia	4(5.3)	0(0)	0.128	0.33	0.01–0.65
Complete right bundle branch block	3(3.9)	3(3.9)	1.000	0.00	−0.32–0.32
First-degree atrioventricular block	3(3.9)	2(2.6)	1.000	0.07	−0.24–0.39
Sinus bradycardia	2(2.6)	1(1.3)	1.000	0.09	−0.22–0.41
Junctional tachycardia	1(1.3)	3(3.9)	0.612	−0.16	−0.48–0.15
Sinus capture	1(1.3)	1(1.3)	1.000	0.00	−0.32–0.32
Ventricular tachycardia	1(1.3)	1(1.3)	1.000	0.00	−0.32–0.32
Supraventricular tachycardia	1(1.3)	0(0)	1.000	0.16	−0.16–0.48

Categorical data are presented as counts (%).

## 4 Discussion

This study represents the first randomized controlled trial to evaluate the preventive effect of early administration of SFI on SIC in adult ICU patients. Previous clinical studies on SFI have primarily focused on the treatment of heart failure, acute coronary syndrome, cardiac arrest, or established SIC. In contrast, this is the first investigation to explore SFI’s role in preventing the onset of SIC. All enrolled patients met the Sepsis 3.0 diagnostic criteria and received standardized care according to the SSC 2021 guidelines, including antimicrobial therapy, fluid resuscitation, vasoactive agents, and organ support, in addition to the assigned intervention. This protocol provides a robust basis for assessing the clinical utility of SFI in SIC prevention. The study findings showed that the overall incidence of SIC within 7 days was 9.2%, which was substantially lower than the expected incidence of 60%. Early administration of SFI did not significantly reduce the incidence of SIC compared with the control group in critically ill adults admitted to the ICU.

The notable discrepancy between the expected and observed incidence of SIC in this study may be attributed to several factors. First, there is currently no universally accepted diagnostic standard for SIC, resulting in considerable variability in diagnostic criteria across studies. This variability is a key reason why reported SIC incidence ranges widely from 10% to 70% (Beesley et al., 2018). In our study, SIC was diagnosed by excluding acute coronary syndrome and stress cardiomyopathy in patients with sepsis, combined with findings of LVEF < 50% and hs-cTNI > 52.4 pg/mL. This criterion is more stringent than those used in prior studies, such as defining SIC by LVEF < 50% or e’<8 cm/s (Landesberg et al., 2012). For example, Landesberg G’s study reported that 63.5% of patients with myocardial dysfunction exhibited isolated diastolic abnormalities. The omission of diastolic function assessment in our study may have led to an underestimation of SIC incidence. Moreover, in our cohort, 55.9% of patients presented with septic shock. Under the premise of “low-resistance” hemodynamic disorder of septic shock. LVEF may overestimate myocardial contractility (Zakynthinos et al., 2025), further limiting the diagnostic effect of LVEF. Second, our center strictly adhered to the SSC 2021 guidelines, ensuring standardized care for all patients. The 3-h sepsis bundle completion rate was approximately 72.3%, and nearly all patients received antimicrobial therapy within 24 h. Early antibiotic administration and rigorous compliance with the sepsis bundle likely enhanced treatment efficacy, contributing to the reduced SIC incidence. This is further evidenced by our observed 28-day overall mortality rate of 25.7%, which is substantially lower than the anticipated 48.59% (Knaus et al., 1985).

NT-proBNP is a protein mainly secreted by ventricular myocytes, which holds significant importance in the diagnosis and monitoring of heart failure and is now widely recommended for use (McDonagh et al., 2021; Heidenreich et al., 2022). In patients with sepsis, NT-proBNP can reflect myocardial injury and is closely related to the prognosis of the disease (Roch et al., 2005; Masson et al., 2016; Vallabhajosyula et al., 2020). Although this study did not demonstrate a significant reduction in SIC incidence with SFI, the observation that NT-proBNP levels were significantly lower in the SFI group compared to the control group after treatment suggests a potential myocardial protective effect of SFI in sepsis patients.

The observed mismatch between the myocardial protective effects of SFI and its lack of significant preventive efficacy against SIC highlights the complexity of SIC pathogenesis. This suggests that modulation of a single therapeutic target may provide limited benefits, and that comprehensive, individualized treatment strategies are essential to effectively address SIC. Additionally, the finding that BUN levels were significantly lower in the SFI group compared to the control group suggests that the multi-mechanistic effects of SFI may also confer benefits to other organ systems. Future clinical studies could be designed to specifically investigate the impact of SFI on the function of other organs in sepsis patients.

SFI has been extensively used in clinical practice for decades across various indications, with few reports of adverse reactions and no serious adverse events documented. Reported occasional adverse reactions include arrhythmias, allergic reactions, medication-related dizziness and headache, and drug-induced liver injury. In this study, we evaluated the safety of continuous early administration of SFI in sepsis patients. The results showed no significant differences between the two groups in the incidence and types of arrhythmia or in the use of antiarrhythmic medications. No cases of allergic reactions, SFI-related neurological dysfunction, or SFI-related liver injury were observed in either group. These findings confirm that the use of SFI in sepsis patients is safe and well-tolerated, consistent with previous research reports (Zhang et al., 2020).

However, this study has several limitations. The actual incidence of SIC observed was 9.2%, which was substantially lower than the expected 60%. This considerable discrepancy resulted in an insufficient sample size to achieve the anticipated statistical power. The primary analysis can only be framed as exploratory rather than confirmatory. Therefore, large-sample randomized controlled trials are still warranted to further validate these findings. What’s more, although the present study has undertaken a series of *post hoc* subgroup analyses, the limited sample size has resulted in an extremely small number of cases in certain subgroups. This has undermined the statistical stability of the findings, so the related analyses can only be exploratory and cannot be regarded as a basis for clinical recommendations. Additionally, some data were missing during the data collection process, which may have affected the robustness of the analyses. To mitigate this, we combined multiple myocardial injury markers and hemodynamic parameters to comprehensively evaluate cardiac function. Moreover, this study primarily focused on the assessment of myocardial injury biomarkers in sepsis that are more conducive to providing mechanistic insights into myocardial protective effects of SFI. In the application of cardiac ultrasound, apart from the LVEF used for diagnosing SIC, no other indicators were subject to strict requirements. During the research process, although we also compared other cardiac ultrasound indicators (Supplementary Table S23 of File S3) of some patients, the results might be biased due to the small sample size and were not included in the analysis. Furthermore, due to the functional allocation of ICU beds at our center, most enrolled patients had severe illness. The mean APACHE II score was 23.21 in the SFI group and 22.66 in the control group, with an overall average of 22.93. Correspondingly, the mean SOFA scores were 9.74 and 10.05, respectively. Some patients may have already progressed beyond the early phase of sepsis, potentially reducing the observable benefits of early intervention with SFI. Future studies focusing on patients with newly diagnosed sepsis are needed to clarify the timing and efficacy of SFI administration. Lastly, this study only captured the incidence of SIC within the first 7 days of ICU admission. As a result, cases of SIC developing after 7 days may have been missed, potentially leading to an underestimation of the true incidence.

## 5 Conclusion

Early application of SFI did not significantly reduce the incidence of SIC in adults in the ICU.

## Data Availability

The original contributions presented in the study are included in the article/Supplementary Material, further inquiries can be directed to the corresponding authors.
