# Supplementary material for: Early administration of shenfu injection for the incidence of sepsis-induced cardiomyopathy in septic patients: a randomized controlled trial
Source: Front Pharmacol. 2026 Feb 27;17:1682246. doi: 10.3389/fphar.2026.1682246 (PMC12982350; doi:10.3389/fphar.2026.1682246)
Supplement: Supplementary file 2 [file DataSheet3.pdf]

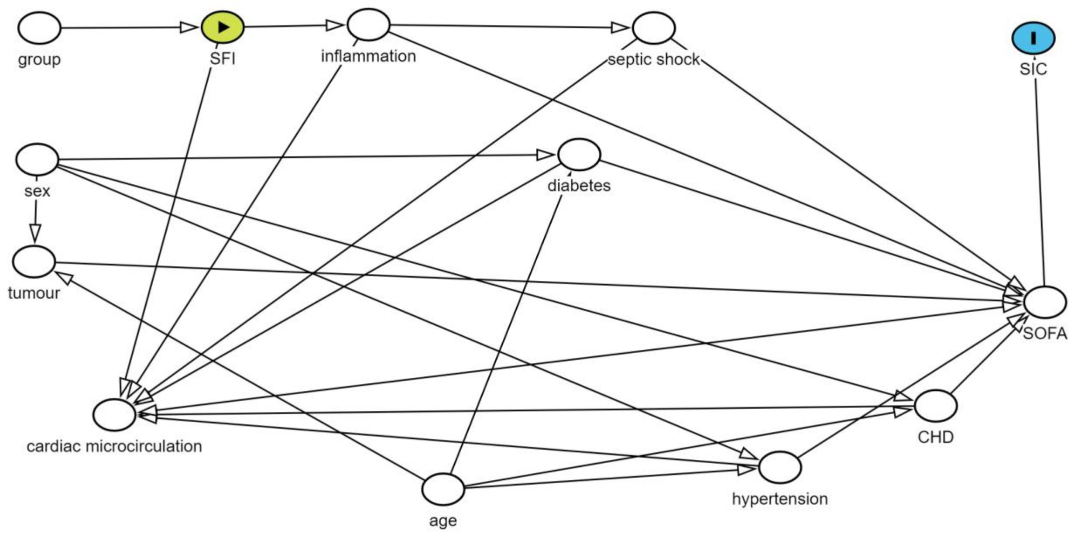

Figure S1 Directed acyclic graph of SFI intervention for SIC

Abbreviations: SFI, shenfu injection; SIC, sepsis-induced cardiomyopathy; CHD, coronary heart disease; SOFA, sequential organ failure assessment

Table S2 Statistical description results of myocardial injury biomarkers

Variable	Time	Group	N	Results	P Value	SMD	95%CI
Hs-cTNI	T0	SFI group	76	44.70（8.90,251.78）			
		Control group	76	53.20（14.20,183.75）			
Hs-cTNI	T1	SFI group	46	24.65（7.93,104.63）			
		Control group	49	23.90（11.65,92.45）			
Hs-cTNI	T2	SFI group	42	13.85（2.65,63.55）			
		Control group	36	29.50（12.90,131.55）			
NT-proBNP	T0	SFI group	57	1748.90（709.60,5419.55）			
		Control group	52	1278.65（564.63,7191.78）			
NT-proBNP	T1	SFI group	50	865.60（150.25,4017.83）			
		Control group	32	777.90（156.53,1924.08）			
NT-proBNP	T2	SFI group	36	988.10（188.38,6449.00）			
		Control group	22	1305.35（176.80,1787.25）			
CK MB	T0	SFI group	75	2.70（1.10,8.20）			
		Control group	75	2.50（0.80,5.50）			
CK MB	T1	SFI group	44	1.40（0.80,4.08）			
		Control group	49	1.40（0.70,2.80）			
CK MB	T2	SFI group	39	1.30（0.70,3.90）			
		Control group	32	1.80（0.60,3.95）			
SOD	T0	SFI group	76	116.36±31.13			
		Control group	76	110.62±26.69			
SOD	T1	SFI group	52	121.23±27.44			
		Control group	47	116.12±23.64			
SOD	T2	SFI group	40	124.47±32.37			
		Control group	37	109.32±19.60			
HMGB 1	T0	SFI group	72	10951.67±1451.94			
		Control group	72	11070.14±1396.02			
HMGB 1	T2	SFI group	40	7314.26±1513.44			
		Control group	44	7716.75±1367.89			
HMGB 1	T0-T2	SFI group	40	3824.97±1985.75	0.497	0.15	-0.28-0.58
		Control group	44	3531.71±1954.40			
H4	T0	SFI group	72	84.07±9.66			
		Control group	72	87.08±9.10			
H4	T2	SFI group	40	61.25±8.54			
		Control group	44	61.72±8.17			
H4	T0-T2	SFI group	40	22.24±12.13	0.145	-0.32	-0.76-0.11
		Control group	44	26.26±11.93			
h-FABP	T0	SFI group	72	2.59±0.69			
		Control group	72	2.66±0.74			
h-FABP	T2	SFI group	40	2.87±0.47			
		Control group	44	2.92±0.48			
h-FABP	T2-T0	SFI group	40	0.23±0.97	0.907	0.03	-0.40-0.45
		Control group	44	0.21±1.03			

Abbreviations:hs-cTNI, high-sensitivity cardiac troponin I;NT-proBNP, N-terminal pro-B-type natriuretic peptide; CKMB, creatine kinase-MB;SOD, superoxide dismutase; HMGB 1, human high mobility group protein B1; h-FABP, heart-type fatty acid binding protein; Human H4, human histone H4.

Table S3 Analysis results of the GLMM model for hs-cTNI

**Model summary**						
Target						hs-cTNI
Probability Distribution						gamma
Link function						Log link function
Information criterion			Akaike corrected			1635.953
			Bayesian			1647.134
**Fixed effects**						
Source	F		df1	df2		Sig.
Corrected Model	8.236		9	315		＜0.001
Group	4.836		1	315		0.029
Times	2.723		2	315		0.067
Group*Time	2.271		2	315		0.105
**Fixed coefficients**						
Model Term	Coefficient	Std.Error	t	Sig	Lower 95%CI	Upper 95%CI
Intercept	5.218	0.4718	11.059	<.001	4.289	6.146
SFI Group	-0.140	0.4940	-0.283	0.778	-1.112	0.832
T2	1.688	0.5854	2.883	0.004	0.536	2.840
T1	0.875	0.4723	1.852	0.065	-0.055	1.804
T2*SFI Group	-1.719	0.8073	-2.129	0.034	-3.307	-0.130
T1*SFI Group	-0.512	0.6755	-0.758	0.449	-1.841	0.817

Adjusted for gender, age, septic shock, and baseline hs-cTNI

Abbreviations:SFI, shenfu injection; sig. significant; df, degree of freedom;CI,confidence interval;hs-cTNI, high-sensitivity cardiac troponin I

Table S4 Analysis results of the GLMM model for NT-proBNP

**Model summary**						
Target				NT-proBNP		
Probability Distribution				gamma		
Link function				Log link function		
Information criterion	Akaike corrected			686.885		
	Bayesian			696.734		
**Fixed effects**						
Source	F		df1	df2		Sig.
Corrected Model	20.332		9	205		<0.001
Group	18.356		1	205		<0.001
Times	2.638		2	205		0.074
Group*Time	5.686		2	205		0.004
**Fixed coefficients**						
Model Term	Coefficient	Std.Error	t	Sig	Lower 95%CI	Upper 95%CI
Intercept	7.158	0.2191	32.674	<0.001	6.726	7.590
SFI Group	0.185	0.2196	0.841	0.401	-0.248	0.618
T2	-0.901	0.3146	-2.863	0.005	-1.521	-0.281
T1	-0.658	0.2512	-2.620	0.009	-1.153	-0.163
T2*SFI Group	1.335	0.4043	3.301	0.001	0.537	2.132
T1*SFI Group	0.588	0.3218	1.827	0.069	-0.047	1.222

Adjusted for gender, age, septic shock, and baseline NT-proBNP

Abbreviations:SFI, shenfu injection; sig., significant; df, degree of freedom;CI,confidence interval; NT-proBNP, N-terminal pro-B-type natriuretic peptide

Table S5 Analysis results of the GLMM model for CK MB

**Model summary**						
Target				CK MB		
Probability Distribution				gamma		
Link function				Log link function		
Information criterion	Akaike corrected			1240.399		
	Bayesian			1251.450		
**Fixed effects**						
Source	F		df1	df2		Sig.
Corrected Model	4.044		9	302		<0.001
Group	0.190		1	302		0.663
Times	5.394		2	302		0.005
Group*Time	0.112		2	302		0.894
**Fixed coefficients**						
Model Term	Coefficient	Std.Error	t	Sig	Lower 95%CI	Upper 95%CI
Intercept	0.988	0.4263	2.316	0.021	0.149	1.826
SFI Group	-0.056	0.4194	-0.134	0.893	-0.882	0.769
T2	-0.533	0.2425	-2.200	0.029	-1.010	-0.056
T1	0.344	0.4680	0.735	0.463	-0.577	1.265
T2*SFI Group	0.031	0.3294	0.093	0.926	-0.617	0.679
T1*SFI Group	-0.302	0.6708	-0.450	0.653	-1.622	1.018

Adjusted for gender, age, septic shock, and baseline CK MB

Abbreviations:SFI, shenfu injection; sig. , significant; df, degree of freedom;CI,confidence interval; CKMB, creatine kinase-MB

Table S6 Analysis results of the LMM model for SOD

**Information criteria**	
-2 log likelihood	2914.39240257
Akaike information criterion	2938.39240257
Akaike information criterion corrected	2939.38287876
Consistent Akaike information criterion	2995.90856587
Bayesian information criterion	2983.90856587

**Fixed effect test**				
Source	df1	df2	F	Sig.
Corrected Model	1	192.908	80.778	<0.001
Group	1	195.506	1.415	0.236
Times	2	249.289	1.618	0.200
Group*Time	2	249.532	0.176	0.838

**Fixed effect estimation**							
Model Term	Estimation	Std.Error	df	t	Sig	Lower 95%CI	Upper 95%CI
Intercept	42.778	5.852	212.290	7.310	<0.001	31.243	54.314
SFI Group	2.272	3.386	322.340	0.671	0.503	-4.390	8.935
T2	2.457	4.131	324.354	0.595	0.552	-5.671	10.585
T1	4.283	3.614	215.210	1.185	0.237	-2.841	11.407
T2*SFI Group	2.924	5.777	323.764	0.506	0.613	-8.442	14.290
T1*SFI Group	-0.457	5.022	212.319	-0.091	0.928	-10.357	9.443

Adjusted for gender, age, septic shock, and baseline SOD

Abbreviations:SFI, shenfu injection; sig. ,significant; df, degree of freedom;CI,confidence interval;SOD, superoxide dismutase

Table S7 Analysis results of the LMM model for HMGB 1

**Information criteria**							
-2 log likelihood	3822.07655993						
Akaike information criterion	3842.07655993						
Akaike information criterion corrected	3843.09038482						
Consistent Akaike information criterion	3886.37001622						
Bayesian information criterion	3876.37001622						
**Fixed effect test**							
Source	df1	df2	F	Sig.			
Corrected Model	1	149.341	19.210	<0.001			
Group	1	141.450	2.535	0.114			
Times	1	160.625	514.418	<0.001			
Group*Time	1	160.667	0.981	0.323			
**Fixed effect estimation**							
Model Term	Estimation	Std.Error	df	t	Sig	Lower 95%CI	Upper 95%CI
Intercept	3984.553	541.349	156.982	7.360	<0.001	2915.285	5053.821
SFI Group	-56.815	179.148	222.134	-0.317	0.751	-409.862	296.231
T2	-3447.700	221.447	157.253	-15.569	<0.001	-3885.094	-3010.305
T2*SFI Group	-314.268	317.316	160.667	-0.990	0.323	-940.915	312.380

Adjusted for gender, age, septic shock, and baseline HMGB1

Abbreviations:SFI, shenfu injection; sig. ,significant; df, degree of freedom;CI,confidence interval;HMGB 1, human high mobility group protein B1

Table S8 Analysis results of the LMM model for h-FABP

**Information criteria**	
-2 log likelihood	283.39440857
Akaike information criterion	303.39440857
Akaike information criterion corrected	304.40823345
Consistent Akaike information criterion	347.68786486
Bayesian information criterion	337.68786486

**Fixed effect test**				
Source	df1	df2	F	Sig.
Corrected Model	1	105.731	357.852	<0.001
Group	1	96.628	0.066	0.798
Times	1	148.806	11.810	<0.001
Group*Time	1	148.686	0.045	0.832

**Fixed effect estimation**							
Model Term	Estimation	Std.Error	df	t	Sig	Lower 95%CI	Upper 95%CI
Intercept	1.583	0.113	163.119	14.044	<0.001	1.361	1.806
SFI Group	-0.029	0.083	176.804	-0.342	0.733	-0.193	0.136
T2	0.259	0.113	145.358	2.298	0.023	0.036	0.482
T2*SFI Group	0.034	0.161	148.686	0.212	0.832	-0.284	0.352

Adjusted for gender, age, septic shock, and baseline h-FABP

Abbreviations:SFI, shenfu injection; sig. ,significant; df, degree of freedom;CI,confidence interval; h-FABP, heart-type fatty acid binding protein

Table S9 Analysis results of the LMM model for H4

**Information criteria**	
-2 log likelihood	1496.07844613
Akaike information criterion	1516.07844613
Akaike information criterion corrected	1517.09227101
Consistent Akaike information criterion	1560.37190242
Bayesian information criterion	1550.37190242

**Fixed effect test**				
Source	df1	df2	F	Sig.
Corrected Model	1	135.440	41.760	<0.001
Group	1	136.339	0.039	0.844
Times	1	160.957	589.724	<0.001
Group*Time	1	160.989	2.389	0.124

**Fixed effect estimation**							
Model Term	Estimation	Std.Error	df	t	Sig	Lower 95%CI	Upper 95%CI
Intercept	35.216	3.533	139.860	9.967	<0.001	28.230	42.201
SFI Group	-1.384	1.109	217.823	-1.248	0.213	-3.569	0.801
T2	-25.756	1.393	157.433	-18.489	<0.001	-28.507	-23.004
T2*SFI Group	3.085	1.996	160.989	1.546	0.124	-0.856	7.027

Adjusted for gender, age, septic shock, and baseline H4

Abbreviations:SFI, shenfu injection; sig. ,significant; df, degree of freedom;CI,confidence interval; Human H4, human histone H4.

Table S10 Statistical description results of hemodynamic parameters

Variable	Time	N	Group	Results
HR	T0	76	SFI group	88.53±18.24
		76	Control group	90.50±17.55
HR	T1	76	SFI group	84.59±17.16
		75	Control group	85.56±15.52
HR	T2	63	SFI group	87.59±15.54
		61	Control group	87.46±15.58
MAP	T0	76	SFI group	84.09±11.12
		76	Control group	84.77±12.86
MAP	T1	76	SFI group	86.11±11.67
		75	Control group	86.27±12.95
MAP	T2	63	SFI group	83.20±11.62
		61	Control group	86.51±15.35
PP	T0	76	SFI group	65.64±17.59
		76	Control group	63.34±21.50
PP	T1	76	SFI group	67.43±18.68
		75	Control group	67.61±17.63
PP	T2	63	SFI group	68.78±17.13
		61	Control group	69.00±18.75
CVP	T0	48	SFI group	9.52±3.58
		45	Control group	9.09±3.21
CVP	T1	48	SFI group	9.69±3.33
		40	Control group	9.09±2.83
CVP	T2	34	SFI group	8.84±2.50
		32	Control group	8.81±2.90
MPP	T0	48	SFI group	74.76±11.88
		45	Control group	76.63±12.69
MPP	T1	48	SFI group	76.58±12.03
		40	Control group	77.45±13.15
MPP	T2	34	SFI group	74.04±12.14
		32	Control group	80.32±16.56
CPP	T0	48	SFI group	53.75±10.66
		45	Control group	56.24±11.16
CPP	T1	48	SFI group	54.31±11.28
		40	Control group	55.96±11.41
CPP	T2	34	SFI group	51.30±10.35
		32	Control group	57.94±13.74
SCVO2	T0	36	SFI group	68.28±16.35
		33	Control group	63.72±12.92
SCVO2	T1	20	SFI group	59.38±11.81
		20	Control group	58.18±12.56
GAP	T0	36	SFI group	6.59±4.15
		33	Control group	7.58±3.26
GAP	T1	20	SFI group	4.75±2.88
		19	Control group	4.92±3.21

Table S10(continued) Statistical description results of hemodynamic parameters

Variable	Time	Group	N	Results	P value	SMD	*95%CI*
Lac≥2mmol/L							
		22	Control group	1.70（1.10,2.93）			
LCR	T1	37	SFI group	61.54（30.50,74.21）	0.468	0.27	-0.21-0.75
		31	Control group	51.43（33.33,72.00）			
LCR	T2	28	SFI group	61.82（45.87,76.97）	0.028	0.56	-0.01-1.13
		22	Control group	47.61（29.55,68.18）			
Lac＜2mmol/L							
Lac	T0	36	SFI group	1.20（0.93,1.60）			
		41	Control group	1.10（0.90,1.30）			
Lac	T1	35	SFI group	1.40（1.10,2.00）			
		40	Control group	1.45（1.03,2.00）			
lac	T2	26	SFI group	1.65（1.10,2.23）			
		32	Control group	1.30（0.93,1.70）			

Abbreviations: HR, heart rate; MAP, mean arterial pressure; PP, pulse pressure; CVP, central venous pressure;MPP; mean perfusion pressure; CPP, coronary perfusion pressure; SCVO2, central venous oxygen saturation; GAP, central venous-to-arterial carbon dioxide difference; lac, lactic acid; lac≥2mmol/L; LCR, lactate clearance.

Table S11 Analysis results of the LMM model for HR

**Information criteria**	
-2 log likelihood	3401.30439784
Akaike information criterion	3407.30439784
Akaike information criterion corrected	3407.36250922
Consistent Akaike information criterion	3422.40365651
Bayesian information criterion	3419.40365651

**Fixed effect test**				
Source	df1	df2	F	Sig.
Corrected Model	1	192.807	120.079	<0.001
Group	1	196.284	0.035	0.852
Times	2	304.365	4.339	0.014
Group*Time	2	304.337	0.353	0.703

**Fixed effect estimation**							
Model Term	Estimation	Std.Error	df	t	Sig	Lower 95%CI	Upper 95%CI
Intercept	41.007	4.098	221.361	10.007	<0.001	32.931	49.083
SFI Group	-1.427	2.202	412.008	-0.648	0.517	-5.755	2.902
T2	-3.193	2.309	416.438	-1.383	0.167	-7.732	1.345
T1	-4.744	2.101	261.566	-2.258	0.025	-8.882	-0.606
T2*SFI Group	2.685	3.251	416.428	0.826	0.409	-3.705	9.075
T1*SFI Group	0.810	2.966	260.899	0.273	0.785	-5.031	6.651

Adjusted for gender, age, septic shock, and baseline HR

Abbreviations:SFI, shenfu injection; sig. ,significant; df, degree of freedom;CI,confidence interval; HR, heart rate

Table S12 Analysis results of the LMM model for MAP

**Information criteria**	
-2 log likelihood	3249.05078761
Akaike information criterion	3255.05078761
Akaike information criterion corrected	3255.10889899
Consistent Akaike information criterion	3270.15004627
Bayesian information criterion	3267.15004627

**Fixed effect test**				
Source	df1	df2	F	Sig.
Corrected Model	1	182.229	108.958	<0.001
Group	1	179.905	0.863	0.354
Times	2	292.242	1.207	0.301
Group*Time	2	292.223	0.939	0.392

**Fixed effect estimation**							
Model Term	Estimation	Std.Error	df	t	Sig	Lower 95%CI	Upper 95%CI
Intercept	43.624	4.536	196.246	9.618	<0.001	34.679	52.569
SFI Group	-0.416	1.845	403.831	-0.226	0.822	-4.043	3.210
T2	1.603	1.922	414.705	0.834	0.405	-2.174	5.380
T1	1.379	1.702	254.828	0.810	0.419	-1.973	4.731
T2*SFI Group	-2.774	2.705	414.662	-1.025	0.306	-8.091	2.543
T1*SFI Group	0.639	2.403	254.148	0.266	0.791	-4.094	5.371

Adjusted for gender, age, septic shock, and baseline MAP

Abbreviations:SFI, shenfu injection; sig. ,significant; df, degree of freedom;CI,confidence interval;MAP, mean arterial pressure

Table S13 Analysis results of the LMM model for PP

**Information criteria**	
-2 log likelihood	3473.35446879
Akaike information criterion	3479.35446879
Akaike information criterion corrected	3479.41258017
Consistent Akaike information criterion	3494.45372746
Bayesian information criterion	3491.45372746

Fixed effect test

Source	df1	df2	F	Sig.
Corrected Model	1	185.324	95.502	<0.001
Group	1	182.698	0.252	0.616
Times	2	295.812	2.993	0.052
Group*Time	2	295.793	0.384	0.681

Fixed effect estimation

Model Term	Estimation	Std.Error	df	t	Sig	Lower 95%CI	Upper 95%CI
Intercept	28.444	3.458	229.347	8.225	<0.001	21.630	35.258
SFI Group	0.802	2.437	391.842	0.329	0.742	-3.988	5.593
T2	5.600	2.501	411.654	2.239	0.026	0.684	10.516
T1	3.760	2.148	266.238	1.750	0.081	-0.469	7.989
T2*SFI Group	-2.961	3.520	411.573	-0.841	0.401	-9.881	3.959
T1*SFI Group	-1.970	3.031	265.584	-0.650	0.516	-7.939	3.998

Adjusted for gender, age, septic shock, and baseline PP

Abbreviations:SFI, shenfu injection; sig. ,significant; df, degree of freedom;CI,confidence interval; PP, pulse pressure

Table S14 Analysis results of the LMM model for CVP

Information criteria

-2 log likelihood	1137.34744755
Akaike information criterion	1143.34744755
Akaike information criterion corrected	1143.45045184
Consistent Akaike information criterion	1156.75162797
Bayesian information criterion	1153.75162797

Fixed effect test

Source	df1	df2	F	Sig.
Corrected Model	1	107.411	54.303	<0.001
Group	1	107.156	0.177	0.675
Times	2	167.767	1.451	0.237
Group*Time	2	167.728	0.122	0.885

Fixed effect estimation

Model Term	Estimation	Std.Error	df	t	Sig	Lower 95%CI	Upper 95%CI
Intercept	3.835	0.639	147.059	6.006	<0.001	2.573	5.097
SFI Group	0.131	0.520	223.543	0.251	0.802	-0.894	1.155
T2	-0.470	0.562	235.077	-0.836	0.404	-1.578	0.638
T1	-0.042	0.485	154.515	-0.087	0.931	-1.000	0.915
T2*SFI Group	-0.139	0.783	234.655	-0.178	0.859	-1.683	1.404
T1*SFI Group	0.207	0.663	151.360	0.312	0.756	-1.103	1.517

Adjusted for gender, age, septic shock, and baseline CVP

Abbreviations:SFI, shenfu injection; sig. ,significant; df, degree of freedom;CI,confidence interval; CVP, central venous pressure

Table S15 Analysis results of the LMM model for MPP

Information criteria	
-2 log likelihood	1876.27957837
Akaike information criterion	1882.27957837
Akaike information criterion corrected	1882.38258266
Consistent Akaike information criterion	1895.68375879
Bayesian information criterion	1892.68375879

Fixed effect test

Source	df1	df2	F	Sig.
Corrected Model	1	112.961	51.491	<0.001
Group	1	106.975	1.339	0.250
Times	2	167.118	0.527	0.591
Group*Time	2	167.187	1.278	0.281

Fixed effect estimation

Model Term	Estimation	Std.Error	df	t	Sig	Lower 95%CI	Upper 95%CI
Intercept	35.538	5.795	121.378	6.132	<0.001	24.065	47.011
SFI Group	-0.381	2.442	229.218	-0.156	0.876	-5.192	4.430
T2	3.697	2.666	236.234	1.387	0.167	-1.556	8.949
T1	1.438	2.359	150.557	0.610	0.543	-3.223	6.100
T2*SFI Group	-4.982	3.716	236.028	-1.341	0.181	-12.304	2.340
T1*SFI Group	0.383	3.229	147.303	0.119	0.906	-5.998	6.765

Adjusted for gender, age, septic shock, and baseline MPP

Table S16 Analysis results of the LMM model for CPP

**Information criteria**	
-2 log likelihood	1784.03633204
Akaike information criterion	1790.03633204
Akaike information criterion corrected	1790.13933633
Consistent Akaike information criterion	1803.44051246
Bayesian information criterion	1800.44051246

**Fixed effect test**				
Source	df1	df2	F	Sig.
Corrected Model	1	113.737	49.513	<0.001
Group	1	110.027	1.779	0.185
Times	2	169.246	0.361	0.697
Group*Time	2	169.442	1.256	0.287

**Fixed effect estimation**							
Model Term	Estimation	Std.Error	df	t	Sig	Lower 95%CI	Upper 95%CI
Intercept	21.234	3.835	127.104	5.537	<0.001	13.645	28.822
SFI Group	-0.460	2.007	232.474	-0.229	0.819	-4.415	3.494
T2	1.263	2.198	236.757	0.574	0.566	-3.068	5.593
T1	0.340	1.983	150.249	0.171	0.864	-3.579	4.259
T2*SFI Group	-4.160	3.064	236.685	-1.358	0.176	-10.197	1.877
T1*SFI Group	0.225	2.716	146.971	0.083	0.934	-5.143	5.592

Adjusted for gender, age, septic shock, and baseline CPP

Abbreviations:SFI, shenfu injection; sig. ,significant; df, degree of freedom;CI,confidence interval; CPP, coronary perfusion pressure

Table S17 Analysis results of the GLMM model for lac＜2mmol/L subgroup

**Model summary**		
Target		Lac
Probability Distribution		gamma
Link function		Log link function
Information criterion	Akaike corrected	256.499
	Bayesian	266.271

**Fixed effects**				
Source	F	df1	df2	Sig.
Corrected Model	8.439	9	200	<0.001
Group	0.007	1	200	0.935
Times	15.598	2	200	<0.001
Group*Time	0.228	2	200	0.796

**Fixed coefficients**						
Model Term	Estimation	Std.Error	t	Sig	Lower 95%CI	Upper 95%CI
Intercept	-0.495	0.1774	-2.788	0.006	-0.845	-0.145
SFI Group	-0.017	0.0981	-0.174	0.862	-0.211	0.176
T2	0.268	0.0975	2.749	0.007	0.076	0.460
T1	0.334	0.0856	3.904	<0.001	0.165	0.503
T2*SFI Group	0.078	0.1443	0.541	0.589	-0.207	0.363
T1*SFI Group	-0.010	0.1252	-0.083	0.934	-0.257	0.236

Adjusted for gender, age, septic shock, and baseline lac

Abbreviations:SFI, shenfu injection; sig. ,significant; df, degree of freedom;CI,confidence interval; lac;lactic acid

Table S18 Analysis results of the GLMM model for lac≥2mmol/L subgroup

**Model summary**		
Target		Lac
Probability Distribution		gamma
Link function		Log link function
Information criterion	Akaike corrected	264.391
	Bayesian	273.885

**Fixed effects**				
Source	F	df1	df2	Sig.
Corrected Model	23.659	9	183	<0.001
Group	1.806	1	183	0.181
Times	67.862	2	183	<0.001
Group*Time	2.724	2	183	0.068

**Fixed coefficients**						
Model Term	Estimation	Std.Error	t	Sig	Lower 95%CI	Upper 95%CI
Intercept	0.919	0.1004	9.152	<0.001	0.721	1.117
SFI Group	0.059	0.1047	0.560	0.576	-0.148	0.265
T2	-0.548	0.1200	-4.568	<0.001	-0.785	-0.311
T1	-0.689	0.1011	-6.812	<0.001	-0.888	-0.489
T2*SFI Group	-0.372	0.1615	-2.301	0.023	-0.690	-0.053
T1*SFI Group	-0.102	0.1375	-0.745	0.457	-0.374	0.169

Adjusted for gender, age, septic shock, and baseline lac

Abbreviations:SFI, shenfu injection; sig. ,significant; df, degree of freedom;CI,confidence interval; lac;lactic acid

Table S19 Analysis results of the LMM model for $SCVO_2$

**Information criteria**	
-2 log likelihood	698.72053106
Akaike information criterion	704.72053106
Akaike information criterion corrected	704.99019398
Consistent Akaike information criterion	715.31832953
Bayesian information criterion	712.31832953

**Fixed effect test**				
Source	df1	df2	F	Sig.
Corrected Model	1	55.777	25.283	<0.001
Group	1	57.892	0.055	0.816
Times	1	76.350	10.539	0.002
Group*Time	1	76.369	1.115	0.294

**Fixed effect estimation**							
Model Term	Estimation	Std.Error	df	t	Sig	Lower 95%CI	Upper 95%CI
Intercept	19.018	3.907	67.298	4.868	<0.001	11.221	26.815
SFI Group	1.728	2.155	92.064	0.802	.0425	-2.552	6.007
T2	-4.443	2.943	76.792	-1.510	0.135	-10.304	1.418
T2*SFI Group	-4.266	4.040	76.369	-1.056	0.294	-12.312	3.780

Adjusted for gender, age, septic shock, and baseline $SCV0_2$

Abbreviations:SFI, shenfu injection; sig. ,significant; df, degree of freedom;CI,confidence interval; SCVO2, central venous oxygen saturation

Table S20 Analysis results of the LMM model for GAP

**Information criteria**	
-2 log likelihood	448.99931556
Akaike information criterion	454.99931556
Akaike information criterion corrected	455.26897848
Consistent Akaike information criterion	465.59711403
Bayesian information criterion	462.59711403

**Fixed effect test**				
Source	df1	df2	F	Sig.
Corrected Model	1	58.253	6.575	0.013
Group	1	55.513	1.324	0.255
Times	1	77.652	23.465	<0.001
Group*Time	1	77.633	2.707	0.104

**Fixed effect estimation**							
Model Term	Estimation	Std.Error	df	t	Sig	Lower 95%CI	Upper 95%CI
Intercept	2.819	0.663	78.510	4.250	<0.001	1.499	4.139
SFI Group	-0.385	0.572	91.325	-0.673	0.503	-1.520	0.751
T2	-3.524	0.789	78.101	-4.468	<0.001	-5.094	-1.954
T2*SFI Group	1.787	1.086	77.633	1.645	0.104	-0.376	3.949

Adjusted for gender, age, septic shock, and baseline GAP

Abbreviations:SFI, shenfu injection; sig. ,significant; df, degree of freedom;CI,confidence interval;GAP, central venous-to-arterial carbon dioxide difference

Table S21 The result of Statistical analysis of immune inflammatory indicators.

Variable	Time	N	Group	results	*P Value*	SMD	95%CI
PCT	T0	76	SFI group	14.16（3.13，63.32）	0.291	0.10	-0.21-0.42
		76	Control group	10.34（1.44，46.89）			
PCT	T1	71	SFI group	4.83（1.20，15.43）	0.259	-0.04	-0.37-0.29
		68	Control group	3.58（0.96，16.56）			
PCT	T2	54	SFI group	2.87（1.12，7.11）	0.278	-0.08	-0.46-0.30
		53	Control group	1.81（0.47，8.30）			
LYM	T0	76	SFI group	0.51（0.23，0.85）	0.894	-0.03	-0.35-0.28
		76	Control group	0.63（0.30，0.89）			
LYM	T1	70	SFI group	0.50（0.30，0.93）	0.669	0.14	-0.20-0.48
		63	Control group	0.50（0.30，0.80）			
LYM	T2	56	SFI group	0.60（0.40，1.00）	0.047	0.36	-0.03-0.75
		48	Control group	0.48（0.30，0.80）			
NEUT	T0	76	SFI group	10.61（6.08，18.45）	0.474	0.07	-0.25-0.39
		76	Control group	10.45（5.44，16.34）			
NEUT	T1	70	SFI group	9.70（6.93，13.53）	0.274	0.27	-0.07-0.61
		63	Control group	9.50（4.80，13.00）			
NEUT	T2	56	SFI group	8.98（6.42，14.20）	0.523	0.27	-0.11-0.66
		48	Control group	8.80（6.14，12.39）			
NLR	T0	76	SFI group	20.20（10.93，38.02）	0.602	-0.16	-0.48-0.16
		76	Control group	19.03（11.72，32.50）			
NLR	T1	70	SFI group	19.30（11.79，29.57）	0.475	-0.12	-0.46-0.22
		63	Control group	16.86（7.75，32.33）			
NLR	T2	56	SFI group	15.40（9.33，22.86）	0.769	-0.31	-0.70-0.08
		48	Control group	15.34（8.20，32.69）			
PLR	T0	76	SFI group	271.50（145.00，442.50）	0.972	-0.24	-0.55-0.08
		76	Control group	268.66（117.56，494.89）			
PLR	T1	70	SFI group	182.29（114.57，276.98）	0.252	-0.34	-0.69-0.00
		63	Control group	205.00（117.86，380.00）			
PLR	T2	56	SFI group	184.06（113.86，321.19）	0.417	-0.18	-0.57-0.20
		48	Control group	214.67（117.58，396.50）			
PCTcT0-T1	T0-T1	71	SFI group	66.78（44.08，80.58）	0.888	-0.13	-0.46-0.20
		68	Control group	67.63（33.78，80.85）			
PCTcT0-T2	T0-T2	54	SFI group	81.96（56.61，91.03）	0.965	0.08	-0.30-0.46
		53	Control group	84.09（46.02，91.35）			
dNLR	T0	76	SFI group	0.96（0.93，0.97）	0.053	0.28	-0.04-0.59
		76	Control	0.94（0.91，0.96）			

Variable	Time	N	Group	results	*P Value*	SMD	95%CI
			group				
dNLR	T1	70	SFI group	0.94（0.91，0.96）	0.271	0.19	-0.15-0.53
		63	Control group	0.94（0.90，0.96）			
dNLR	T2	56	SFI group	0.93（0.90，0.96）	0.927	0.14	-0.25-0.52
		48	Control group	0.92（0.88，0.96）			
SII	T0	76	SFI group	2187.78（1409.02，5323.75）	0.874	-0.04	-0.36-0.27
		76	Control group	2490.20（1271.04，5277.53）			
SII	T1	70	SFI group	1789.76（899.07，3182.39）	0.951	-0.20	-0.54-0.14
		63	Control group	1681.33（837.00，3567.57）			
SII	T2	56	SFI group	1606.36（945.43，3395.55）	0.725	-0.20	-0.59-0.18
		48	Control group	2262.02（756.69，4254.50）			

Abbreviations: PCT, procalcitonin; NEUT, neutrophil; LYM, lymphocyte; PCTc, procalcitonin clearance; NLR, neutrophil-to- lymphocyte ratio; PLR, platelet-to- lymphocyte rate; dNLR, neutrophil / (leukocyte minus lymphocyte); SII, systemic immune-inflammation (platelet* neutrophil / lymphocyte).

Table S22 The result of Statistical analysis of organ function.

Variable	Time	N	Group	Results	P Value	SMD	95%CI
PLT	T0	76	SFI group	144.50（76.00，215.75）	0.631	0.15	-0.17-0.47
		76	Control group	123.00（73.25，190.75）			
PLT	T1	70	SFI group	106.00（53.50，171.75）	0.734	0.04	-0.30-0.38
		63	Control group	104.00（61.00，167.00）			
PLT	T2	56	SFI group	121.50（78.00，214.50）	0.276	0.29	-0.09-0.68
		48	Control group	114.00（66.00，156.00）			
TB	T0	75	SFI group	22.50（11.30，39.40）	0.515	0.11	-0.21-0.43
		76	Control group	19.40（11.15，36.53）			
TB	T1	57	SFI group	21.50（10.65，50.95）	0.167	0.29	-0.09-0.66
		53	Control group	14.70（8.55，34.55）			
TB	T2	36	SFI group	21.90（11.88，43.25）	0.310	0.18	-0.27-0.63
		41	Control group	18.00（10.40，28.55）			
AST	T0	76	SFI group	55.00（31.50，137.00）	0.588	0.15	-0.17-0.46
		76	Control group	55.00（28.00，126.00）			
AST	T1	54	SFI group	61.50（30.75，107.25）	0.548	-0.07	-0.45-0.30
		53	Control group	58.00（28.50，83.00）			
AST	T2	36	SFI group	64.00（27.25，124.75）	0.388	0.14	-0.31-0.58
		41	Control group	48.00（29.50，65.00）			
CREA	T0	76	SFI group	112.75（73.20，227.63）	0.921	-0.09	-0.41-0.23
		76	Control group	113.45（67.25，293.13）			
CREA	T1	55	SFI group	105.50（64.80，168.00）	0.328	-0.19	-0.57-0.19
		54	Control group	114.40（68.75，199.15）			
CREA	T2	38	SFI group	109.15（53.60，150.03）	0.120	-0.35	-0.79-0.08
		44	Control group	122.45（72.40，194.00）			
BUN	T0	76	SFI group	12.63.（8.19，22.07）	0.924	-0.11	-0.42-0.21
		76	Control group	12.00（7.20，22.98）			
BUN	T1	55	SFI group	10.30（8.10，15.50）	0.202	-0.25	-0.63-0.12
		55	Control group	13.20（8.93，19.60）			
BUN	T2	38	SFI group	9.74（5.39，17.80）	0.047	-0.40	-0.84-0.04
		44	Control group	13.56（9.17，21.38）			

Abbreviations: PLT, platelet; T, total bilirubin; AST, aspartic transaminase; CREA; creatinine; BUN, urea nitrogen.

Table S23 Echocardiography at T2

Variable	SFI group(n=14)	Control group(n=19)	*P Value*	SMD	95%CI
LAVI	31.09±14.66	34.53±16.38	0.548	-0.23	-0.94-0.48
RVOT-FS	45.79±13.95	46.11±13.85	0.948	-0.02	-0.72-0.67
RVOT-D	22.89±3.05	22.13±3.57	0.526	0.24	-0.46-0.93
MAPSE	16.58±2.68	14.78±4.25	0.161	0.52	-0.16-1.20
TAPSE	20.67±4.84	19.42±5.50	0.486	0.25	-0.42-0.92
VTI	21.41±6.21	21.55±6.07	0.945	-0.02	-0.67-0.62
E/A	0.84（0.60，1.43）	0.87（0.75，1.12）	0.570	0.12	-0.59-0.83
E/e’	8.01±2.17	9.39±2.55	0.116	-0.60	-1.32-0.11
RV S’	16.23±4.44	15.11±5.42	0.540	0.23	-0.47-0.94
RV Dd	5.66±1.92	5.84±1.56	0.772	-0.10	-0.80-0.59

Abbreviations: LAVI, Left atrial volume index; RVOT-FS, Right ventricular outflow tract fractional shortening; RVOT-D, right ventricular outflow tract diameter; MAPSE, Mitral annular plane systolic excursion; TAPSE, Tricuspid annular plane systolic excursion; VTI, Velocity time integral; E/A, Early diastolic mitral inflow velocity / Atrial systolic mitral inflow velocity; E/e’, Early diastolic mitral inflow velocity / Early diastolic mitral annular tissue velocity; RV S’ , Right ventricular tricuspid annular S’ wave peak velocity; RV Dd, Right ventricular diastolic dimension.

Table S24 The reasons for the absences of ultrasound indicators

reason	LAVI	RVOT-FS	RVOT-D	MAPSE	TAPSE	VTI	E/A	E/e’	RV S’	RV Dd
ICU stay less than 7 days	48	48	48	48	48	48	48	48	48	48
Pulmonary air artifact	20	20	20	20	20	20	20	20	20	20
arrhythmia	15	20	14	20	20	22	17	20	16	16
Obesity	10	10	10	10	10	10	10	10	10	10
Diaphragm elevation	10	10	10	10	10	10	10	10	10	10
emphysema	5	5	5	5	5	5	5	5	5	5
pneumoderm	3	3	3	3	3	3	3	3	3	3
pleural effusion	5	5	5	5	5	5	5	5	5	5
Poor coordination	5	5	6	6	5	4	7	5	5	5
hyperdynamic circulation	0	0	0	0	3	3	3	3	3	0
hydropericardium	2	2	2	2	2	2	2	2	2	2
pleural thickening	2	2	2	2	2	2	2	2	2	2
thoracocyllosis	1	1	1	1	1	1	1	1	1	1

Variable	SFI group	Control group	OR（95% CI）	P value	P for interaction
All patients	18/76	21/76	0.813 (0.392~1.686)	0.578	
Sex					
Male	12/44	17/52	0.772 (0.320~1.863)	0.565	0.633
Female	6/32	4/24	1.154 (0.287~4.646)	0.840	
Age					
＜65 yr	8/37	8/37	1.000 (0.331~3.025)	1.000	0.622
＞65 yr	10/39	13/39	0.690 (0.259~1.837)	0.457	
Version of the vasoactive-inotropic score					
＜15	11/52	17/61	0.694 (0.291~1.657)	0.411	0.570
≥15	7/24	4/15	1.132 (0.267~4.797)	0.866	
Noradrenaline equivalent					
＜0.1	9/42	11/48	0.917 (0.338~2.489)	0.865	0.644
≥0.1	9/34	10/28	0.648 (0.219~1.918)	0.433	
Hypertension					
No	10/46	13/38	0.534 (0.203~1.409)	0.205	0.216
Yes	8/30	8/38	1.364 (0.443~4.195)	0.589	
Diabetes					
No	15/56	17/54	0.796 (0.349~1.816)	0.588	0.998
Yes	3/20	4/22	0.794 (0.154~4.082)	0.783	
Malignant tumor					
No	11/56	8/55	1.436 (0.529~3.897)	0.477	0.076
Yes	7/20	13/21	0.331 (0.093~1.183)	0.089	
Lac					
＜2mmol/L	7/36	13/41	0.520 (0.181~1.494）	0.224	0.236
≥2mmol/L	11/40	8/35	1.280 (0.448~3.661)	0.645	
Septic shock					
No	4/33	13/44	0.329 (0.096~1.125)	0.076	0.069
Yes	14/43	8/32	1.448 (0.521~4.030)	0.478	

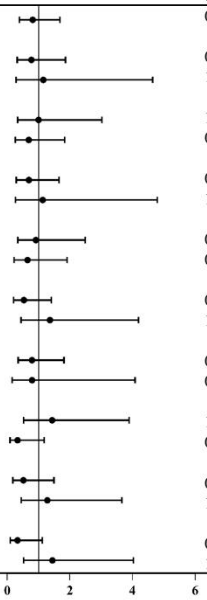

Figure S2 Forest Plot of Subgroup Analysis for 28-day Mortality Based on Binary Logistic Regression (Including P-value for Interaction)

Notes: OR＜1 means SFI Group better, OR＞1 means Control Group better,
