# Supplementary material for: Early administration of shenfu injection for the incidence of sepsis-induced cardiomyopathy in septic patients: a randomized controlled trial
Source: Front Pharmacol. 2026 Feb 27;17:1682246. doi: 10.3389/fphar.2026.1682246 (PMC12982350; doi:10.3389/fphar.2026.1682246)
Supplement: Supplementary file 3 [file DataSheet1.pdf]

Product information of Shenfu Injection

Generic name: Shenfu Injection

Manufacture: China Resources Sanjiu Pharmaceutical Co., Ltd.

Approval number: Z51020664

Batch number:230604AK01

Specification: 10ml per bottle

Table S1 Quality analysis of Shenfu injection* (Batch number:230604AK01)

Item	Content	Quality control standard
Consistency	0.9	≥0.9
Total saponin	1.0 mg/mL	0.7~1.7 mg/mL
Ginsenoside Rg1	0.1 mg/mL	≥0.08 mg/mL
Ginsenoside Re	0.1 mg/mL	≥0.06 mg/mL

*The data is sourced from the product inspection report of China Resources Sanjiu Pharmaceutical Co., Ltd.

Computational formula

NEE=Norepinephrine(μg/kg/min)+Epinephrine(μg/kg/min)+1/10×phenylephrine(μg/kg/min)+1/100×dopamine(μg/kg/min)+1/8×aramine(μg/kg/min)+2.5×vasopressin(U/min)+10×Angiotensin II(μg/kg/min)

VIS (Gaies Version)=dopamine(μg/kg/min)+dobutamine(μg/kg/min)+100×Epinephrine(μg/kg/min)+100×Norepinephrine (μg/kg/min)+10000×vasopressin(U/min)+10×mililong(μg/kg/min)

VIS 2021 Edition (Belletti Version)=VIS(Gaies Version)+Enoximone(μg/kg/min)+50×Levosimendan(μg/kg/min)+25×Olprinone(μg/kg/min)+20×methylene blue(mg/kg/h)+10×phenylephrine(μg/kg/min)+10×terlipressin(μg/min)+0.25×Angiotensin II(ng/kg/min)
